# Genome Sequence of *Dyella ginsengisoli* Strain LA-4, an Efficient Degrader of Aromatic Compounds

**DOI:** 10.1128/genomeA.00961-13

**Published:** 2013-11-21

**Authors:** Chunlei Kong, Lijuan Wang, Pengpeng Li, Yuanyuan Qu, Hongzhi Tang, Jingwei Wang, Hao Zhou, Qiao Ma, Jiti Zhou, Ping Xu

**Affiliations:** State Key Laboratory of Industrial Ecology and Environmental Engineering (MOE), School of Environmental Science and Technology, Dalian University of Technology, Dalian, Chinaa; State Key Laboratory of Microbial Metabolism and School of Life Sciences & Biotechnology, Shanghai Jiao Tong University, Shanghai, Chinab

## Abstract

*Dyella ginsengisoli* strain LA-4 can efficiently degrade environmental pollutants such as biphenyl and azo dyes. Here, we present a 4.55-Mb draft genome sequence of strain LA-4, which may provide further insights into the molecular mechanism in environmental pollution remediation.

## GENOME ANNOUNCEMENT

Microorganisms have been widely investigated in regard to their roles in the degradation of environmental pollutants such as biphenyl and azo dyes, which are carcinogenic and toxic to the central and peripheral nervous systems (1, 2). However, until now, strains of the genus *Dyella* have been rarely reported to be involved in any degradation processes, especially associated with the biodegradation of aromatic compounds. *Dyella ginsengisoli* strain LA-4 was isolated from activated sludge and proven to be an efficient biphenyl-degrading bacterium (3). It also removed azo dyes (acid red GR) and heavy metals (Cu^2+^ and Ni^2+^) (4). The complete 12,186-bp *bphABCXD* gene cluster responsible for biphenyl degradation was successfully cloned and expressed in *Escherichia coli* (3). A novel *meta*-fission product hydrolase gene encoding the lower pathway of biphenyl degradation was located in the *bphX* region (5). Saturation mutagenesis of the *meta*-cleavage product hydrolase BphD reveals that a non-active-site residue, Met148, plays a key role in the catalytic efficiency (6). In addition, BphD demonstrates promiscuous esterase activity toward *p*-nitrophenyl esters (7). These characteristics of strain LA-4 may play important roles in bioremediation of serious pollutants. To date, no genome of a *Dyella ginsengisoli* strain has been sequenced; thus, this draft genome sequence report of strain LA-4 may provide more comprehensive genetic information for the application of this bacterium in environmental pollution remediation in the future.

Here we report the draft genome sequence of *Dyella ginsengisoli* LA-4, which was obtained using Illumina High-Seq 2000 paired-end sequencing, and assembled into 99 contigs using Velvet software 1.2.03 (8). Annotation was performed using the RAST server (9) and the NCBI Prokaryotic Genome Automatic Annotation Pipeline. The contigs were searched against the KEGG and Clusters of Orthologous Groups (COG) databases to analyze the gene functions and metabolic pathways (10). The genome of strain LA-4 has a G+C content of 67.7%. It has 3 rRNA operators, with 1 5S RNA and 50 tRNA genes, and contains 4,099 protein-coding sequences (CDS). Moreover, 124 CDS encoding membrane transport systems were annotated, which may contribute to transporting toxicants of strain LA-4, and 32 proteins involved in the metabolism of aromatic compounds were predicted, which may explain its ability to degrade biphenyl.

The continuous biphenyl metabolism clusters bphA1A2(orf1)A3A4BCX0 and bphX1(orf2)X2X3D were found in a small contig, which has only about 54 kb with 60.4% GC content and some mobile element proteins. Thus, we propose that the contig may be a plasmid, or the cluster may be in the LA-4 genome just by horizontal gene transfer. In addition, a transcriptional regulator protein (GntR family) was found in front of the cluster, which may have a key role in the regulation of the metabolism of biphenyl degradation.

### Nucleotide sequence accession numbers.

The whole-genome shotgun project has been deposited at DDBJ/EMBL/GenBank under the accession number AMSF00000000. The version described in this paper is the first version, AMSF01000000.
